# TALK OF AGES AND EMPTY BOWLS: A RECIPROCAL APPROACH TO INTERGENERATIONAL SERVICE

**DOI:** 10.1093/geroni/igae098.1639

**Published:** 2024-12-31

**Authors:** Joann Montepare

**Affiliations:** Lasell University, Newton, Massachusetts, United States

## Abstract

Contemporary age-inclusive campus initiatives in higher education (Age-Friendly University/AFU, Age Inclusivity Domains of Higher Education/AIDHE) advocate for promoting intergenerational exchange to facilitate the reciprocal sharing of expertise between learners of all ages. This presentation will describe these initiatives as background for the symposium. In addition, it will describe how Lasell University’s “Talk of Ages” program, which takes a “common ground” approach for bringing younger and older learners together in the beyond the classroom, offers a pedagogical philosophy for intergenerational exchange. To demonstrate the value of this approach, we will describe a campus-wide “Empty Bowls” project in which students and older adult community members worked together to craft bowls and to raise money in the mission to fight hunger in the local community. Logistics will be discussed about mounting intergenerational activities as part of this intergenerational service project including offering intergenerational classes in Ceramics, organizing sessions for bowl crafting and glazing, and hosting an intergenerational event where community members come together to enjoy a simple meal of soup and bread in a bowl they select and buy with the proceeds benefitting the local community food pantry. In addition to demonstrating the value of intergenerational collaboration and service as part of being an age-inclusive campus, challenges will be discussed so that educators are better prepared to design related efforts.

